# Supplementary material for: Development of a computer-aided design software for the quantitative evaluation of aesthetic damage
Source: PLoS One. 2019 Dec 18;14(12):e0226322. doi: 10.1371/journal.pone.0226322 (PMC6919621; doi:10.1371/journal.pone.0226322)
Supplement: S1 Code site — 1. (ZIP) [file pone.0226322.s001.zip › [EESC_jr][Projeto]Relatorio_07_21/reports/templates_zip/gentelella-master.zip!/gentelella-master/production/inbox.html]

Gentellela Alela! | 


Gentellela Alela!

Welcome,

## John Doe

  


### General

- Home 
  - Dashboard
  - Dashboard2
  - Dashboard3
- Forms 
  - General Form
  - Advanced Components
  - Form Validation
  - Form Wizard
  - Form Upload
  - Form Buttons
- UI Elements 
  - General Elements
  - Media Gallery
  - Typography
  - Icons
  - Glyphicons
  - Widgets
  - Invoice
  - Inbox
  - Calendar
- Tables 
  - Tables
  - Table Dynamic
- Data Presentation 
  - Chart JS
  - Chart JS2
  - Moris JS
  - ECharts
  - Other Charts
- Layouts 
  - Fixed Sidebar
  - Fixed Footer

### Live On

- Additional Pages 
  - E-commerce
  - Projects
  - Project Detail
  - Contacts
  - Profile
- Extras 
  - 403 Error
  - 404 Error
  - 500 Error
  - Plain Page
  - Login Page
  - Pricing Tables
- Multilevel Menu 
  - Level One- Level One
      - Level Two
      - Level Two
      - Level Two
    - Level One
- Landing Page Coming Soon

- John Doe
  - Profile
  - 50%
    Settings
  - Help
  - Log Out
- 6
  - John Smith
    3 mins ago

    Film festivals used to be do-or-die moments for movie makers. They were where...
  - John Smith
    3 mins ago

    Film festivals used to be do-or-die moments for movie makers. They were where...
  - John Smith
    3 mins ago

    Film festivals used to be do-or-die moments for movie makers. They were where...
  - John Smith
    3 mins ago

    Film festivals used to be do-or-die moments for movie makers. They were where...
  - **See All Alerts**


### Inbox Design Some examples to get you started

Go!

## Inbox DesignUser Mail

- - Settings 1
  - Settings 2

COMPOSE


### Dennis Mugo 3.00 PM

Ut enim ad minim veniam, quis nostrud exercitation enim ad minim veniam, quis nostrud exercitation...


### Jane Nobert 4.09 PM

To Ut enim ad minim veniam, quis nostrud exercitation enim ad minim veniam, quis nostrud exercitation...


### Musimbi Anne 4.09 PM

CC Ut enim ad minim veniam, quis nostrud exercitation enim ad minim veniam, quis nostrud exercitation...


### Jon Dibbs 4.09 PM

Ut enim ad minim veniam, quis nostrud exercitation enim ad minim veniam, quis nostrud exercitation...


.

### Debbis & Raymond 4.09 PM

Ut enim ad minim veniam, quis nostrud exercitation enim ad minim veniam, quis nostrud exercitation...


.

### Debbis & Raymond 4.09 PM

Ut enim ad minim veniam, quis nostrud exercitation enim ad minim veniam, quis nostrud exercitation...


### Dennis Mugo 3.00 PM

Ut enim ad minim veniam, quis nostrud exercitation enim ad minim veniam, quis nostrud exercitation...


### Jane Nobert 4.09 PM

Ut enim ad minim veniam, quis nostrud exercitation enim ad minim veniam, quis nostrud exercitation...


Reply

8:02 PM 12 FEB 2014

#### Donec vitae leo at sem lobortis porttitor eu consequat risus. Mauris sed congue orci. Donec ultrices faucibus rutrum.

**Jon Doe**
(jon.doe@gmail.com) to
**me**

Lorem ipsum dolor sit amet, consectetur adipiscing elit, sed do eiusmod tempor incididunt ut labore et dolore magna aliqua. Ut enim ad minim veniam, quis nostrud exercitation ullamco laboris nisi ut aliquip ex ea commodo consequat.
Duis aute irure dolor in reprehenderit in voluptate velit esse cillum dolore eu fugiat nulla pariatur. Excepteur sint occaecat cupidatat non proident, sunt in culpa qui officia deserunt mollit anim id est laborum.

Riusmod tempor incididunt ut labor erem ipsum dolor sit amet, consectetur adipiscing elit, sed do eiusmod tempor incididunt ut labore et dolore magna aliqua. Ut enim ad minim veniam, quis nostrud exercitation ullamco laboris
nisi ut aliquip ex ea commodo consequat. Duis aute irure dolor in reprehenderit in voluptate velit esse cillum dolore eu fugiat nulla pariatur. Excepteur sint occaecat cupidatat non proident, sunt in culpa qui officia deserunt
mollit anim id est laborum.

Modesed do eiusmod tempor incididunt ut labore et dolore magna aliqua. Ut enim ad minim veniam, quis nostrud exercitation ullamco laboris nisi ut aliquip ex ea commodo consequat. Duis aute irure dolor in reprehenderit in voluptate
velit esse cillum dolore eu fugiat nulla pariatur. Excepteur sint occaecat cupidatat non proident, sunt in culpa qui officia deserunt mollit anim id est laborum.

3 attachments — 
Download all attachments |
View all images

- image-name.jpg

  12KB

  View -
  Download
- img\_name.jpg

  40KB

  View -
  Download
- img\_name.jpg

  30KB

  View -
  Download

Reply


Gentelella - Bootstrap Admin Template by Colorlib

New Message
×

- Huge
- Normal
- Small

Add

Send


>
